# Updates, Management, and Future of Diagnosing and Managing Chronic Lung Allograft Dysfunction

**DOI:** 10.3390/jcm15041543

**Published:** 2026-02-15

**Authors:** Emily Gosche, Joshua B. Smith

**Affiliations:** Division of Pulmonary, Allergy, and Critical Care Medicine, University of Colorado School of Medicine, Aurora, CO 80045, USA; emily.gosche@cuanschutz.edu

**Keywords:** lung transplantation, rejection, chronic lung allograft dysfunction, bronchiolitis obliterans syndrome, management

## Abstract

Lung transplantation provides a curative option for patients living with end-stage lung disease, with a goal of improving survival and quality of life. Chronic lung allograft dysfunction, or CLAD, represents a major cause of morbidity and mortality, particularly after the first year of transplant. **Background/Objectives**: The goal of this review is to outline the diagnosis and management of CLAD within the lung transplant population, as well as discuss future areas of potential research interest. **Methods**: A PubMed literature review of relevant publications regarding CLAD epidemiology, diagnosis, and management was performed to assess current understandings. **Results**: CLAD is the leading cause of death in lung transplant patients following the first year of transplant, and is common, with approximately 50% of patients exhibiting some degree of CLAD within five years of surgery. Well-established guidelines on diagnosis were recently published to aid clinicians in diagnosing and characterizing CLAD. Several medical and surgical interventions exist, although no therapy consistently and reliably stabilizes or reverses CLAD. **Conclusions**: CLAD management remains a priority within the lung transplant field as a leading cause of morbidity and mortality.

## 1. Introduction

Lung transplantation is a curative option for many patients living with end-stage lung disease, and has the largest proportional growth of any solid organ transplant [1]. Long-term survival of lung transplant (LTx) recipients is complicated by chronic lung allograft dysfunction (CLAD) and is the shortest of all solid organ transplants, with a median survival of around 6.5 years [2]. CLAD, an immune-mediated damage to the lungs, is the leading cause of death after the first year of transplantation [2] and is a significant barrier to long-term post-transplant survival. In this review, we will discuss epidemiology, diagnosis, management, and future areas of research interest in CLAD.

## 2. Materials and Methods

This review summarizes the epidemiology, diagnosis, and management of CLAD with a focus on outlining current and prospective management strategies.

A comprehensive literature search was performed using PubMed/MEDLINE to identify relevant publications through January 2026. Considerations were given to recent studies, and priority was given to research studies; however, in many instances, guideline/consensus publications are included due to relevance. When available, multi-center, prospective, and/or randomized controlled trials are given priority; however, many studies cited are single-center, retrospective, and uncontrolled, due to the nature of lung transplant research (significant heterogeneity among center practices limits protocolization and there is a lack of clinical equipoise in many circumstances). Priority was given to studies published within 10 years, although in some circumstances, older papers of significance were included. Approximately 250 publications were screened for inclusion by the authors. Search terms included keywords and controlled vocabulary, including lung transplantation, chronic rejection, chronic lung allograft dysfunction, CLAD, bronchiolitis obliterans syndrome, survival, outcomes, immunosuppression, treatment, management, gastroesophageal reflux, fundoplication, and advances. Searches were limited to human studies in adults, and only studies available in English were included. The reference list of relevant studies and review articles was manually reviewed to identify other potential publications of interest, and both authors independently performed reviews of cited publications for integrity, risk of bias, and relevance. PRISMA guidelines for literature were utilized. This review article does not feature any unpublished data or independent research and does not require institutional review board approval. Generative augmented intelligence was not used in the preparation of this review.

## 3. Results

Although there have been multiple changes to the definition of chronic lung allograft dysfunction over the past several decades, data from the International Society for Heart and Lung Transplantation (ISHLT) in 2018 show that approximately half of adult lung transplant recipients have developed CLAD by five years post-transplant [2]. Kulkarni and colleagues retrospectively assessed the ISHLT registry outcomes for CLAD-free survival from 1994 through 2011 and found a median CLAD-free survival of 3.16 years and 3.58 years for single and bilateral lung recipients, respectively [3]. There are numerous immunologic and non-immunologic risk factors that have been associated with CLAD. Episodes of acute cellular rejection (ACR) [4,5], new donor specific antibodies (DSAs) [6,7], HLA antibodies to self-antigens (e.g., K-alpha 1 tubulin, collagen V) [8], gastroesophageal reflux disease (GERD) [9,10,11], infections [12,13,14], primary graft dysfunction [15], and air pollution [16,17] can all predispose lung transplants recipients to chronic rejection [18,19]. Patient-specific factors, such as telomeropathies, are also associated with decreased CLAD-free survival [20].

### 3.1. CLAD Diagnosis

Chronic rejection has been a known entity in lung transplant literature for decades and historically was referred to as bronchiolitis obliterans syndrome (BOS) until around 2010, as there was growing acknowledgement of other phenotypes [21,22], with different physiology and radiography, including restrictive allograft syndrome (RAS). In 2019, the ISHLT released guidelines to aid in the diagnosis and characterization of CLAD [23].

**Figure 1:**
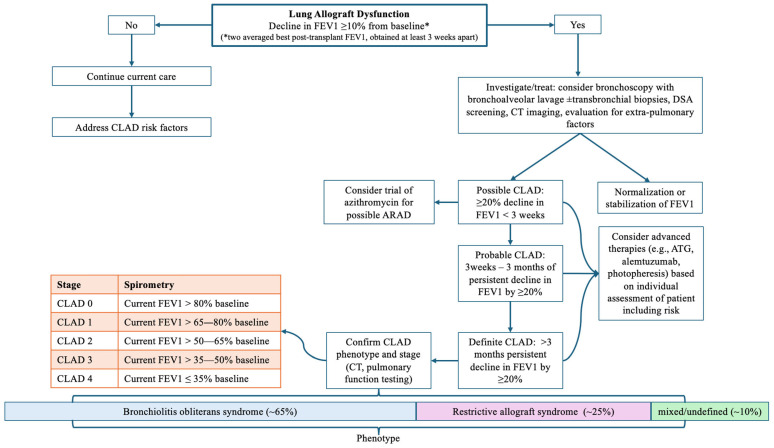
Diagnostic algorithm for CLAD.

Unlike other forms of lung transplant rejection, CLAD is considered a syndrome and is diagnosed based on the patient’s pulmonary function testing, then further characterized by physiology and radiography [23]. Importantly, pathology is not integral to the diagnosis of CLAD and is typically acquired to evaluate for alternative etiologies of graft dysfunction. Subsequently, CLAD is further staged based on a decrease in spirometry. Characterization of CLAD is significant as there are implications with regard to prognosis, as well as considerations when re-transplantation is being considered for a patient [24,25,26]. Figure 1 outlines the diagnostic schema of CLAD, including staging.

The key element in recognizing CLAD is a durable decline in lung function, based on spirometry, ongoing for at least three months. To establish a patient’s post-transplant baseline, their two highest forced expiratory volume at 1 s (FEV1), measured at least three weeks apart, are averaged together to establish the patient’s “baseline” allograft function. At clinic visits, spirometry is performed routinely as part of graft assessment; in many centers, full pulmonary function testing may not be routinely performed due to time constraints. Some variability in measurement is anticipated, but a fluctuation of a ≥10% decline in baseline FEV1 warrants further scrutiny for allograft dysfunction. Investigations are at the discretion of the transplant pulmonology team but typically include bronchoscopy, cross-sectional imaging, and evaluation for the presence of donor-specific antibodies (DSA). It is key to rule out alternative etiologies for graft dysfunction, including acute cellular rejection (ACR), antibody-mediated rejection (AMR), infection, significant weight gain, pleural effusion, lung resection, thoracic cage injuries, and other organ dysfunction that can impact spirometry (e.g., congestive heart failure, neuromuscular disease) [23]. When identified, if reversible, these contributing factors are addressed, such as drainage of pleural effusion or reduction in weight; otherwise, in irreversible pathologies, such as in cases of lung resection, the averaged best FEV1 is recalculated using newly obtained values. Once the patient’s FEV1 falls to ≤80% of baseline, this is considered possible CLAD. At or before this point in a patient’s trajectory, many transplant providers will strongly consider the addition of macrolide therapy, as a small subset of patients may exhibit reversal of graft dysfunction, an entity known in the literature as either neutrophilic reversible allograft dysfunction (NRAD) or azithromycin-responsive allograft dysfunction (ARAD) [27,28]. After three months of durable FEV1 loss ≥20% from baseline, the diagnosis of CLAD is made and is then further characterized by phenotype.

BOS represents a majority of CLAD diagnoses, with approximately two-thirds of patients with CLAD exhibiting this phenotype. Pathology, if obtained, may identify characteristic obliterative bronchiolitis lesions, represented by terminal airway fibrosis and narrowing. These lesions are patchy and can be missed on biopsies. Therefore, the presence of obliterative bronchiolitis lesions does not fall in the diagnostic algorithm of CLAD [29] and is not required for diagnosis, but can support the CLAD diagnosis when present. Patients will demonstrate obstructive physiology on spirometry, with an FEV1/FVC ratio of <70%, and without evidence of restriction, which is identified as a ≥10% reduction in total lung capacity (TLC) when compared to baseline. CT imaging may demonstrate features of air-trapping, mosaic attenuation, centrilobular nodules, and bronchiectasis [30].

The second major phenotype of CLAD is RAS. This is associated with a poorer prognosis and shorter survival when compared to BOS, with a median survival of 6–18 months for RAS compared to 3–5 years for BOS [22]. RAS is characterized by a restrictive pattern on spirometry (FEV1/FVC > 70%, reduction in TLC by ≥10% from baseline). From a practical standpoint, many centers do not routinely perform TLC measurements, so a reduction in FVC by ≥20% from baseline has been proposed as a surrogate [31,32]. Transbronchial biopsies may also identify obliterative bronchiolitis lesions, which can be present in RAS as well, but are not required for diagnosis. Typical radiographic features include the presence of both parenchymal and pleural fibrosis, volume loss, and consolidative opacities. A small number of patients may exhibit characteristics compatible with CLAD, but that do not fit into BOS or RAS phenotyping, which is designated as mixed or undefined phenotypes.

Upon diagnosing and phenotyping CLAD, another significant element is staging CLAD. This is done by evaluating the degree of loss in FEV1 compared to baseline. Stage 0 CLAD is commonly used to describe individuals who have yet to show significant spirometry changes but have obliterative bronchiolitis lesions on pathology; their FEV1 is >80% of baseline. Stage 1 CLAD is for individuals who are ≤80% of baseline function but >65%, stage 2 is for individuals between 65–50%, stage 3 describes patients whose allograft function is between 50–35%, and stage 4 represents the most severe category, in patients with ≤35% function based on FEV1 when compared to baseline. As expected, a higher stage of CLAD portends a poorer prognosis.

One caveat to the current paradigm for characterizing CLAD is the applicability to single-lung transplant recipients, which accounts for approximately a quarter of transplants performed. Due to the presence of native lung dysfunction, spirometry may not accurately represent the underlying pathology in CLAD. Berra and colleagues sought to apply the 2019 diagnostic schema to single-lung transplant recipients [33]. They found moderate interobserver agreement in the diagnosis of CLAD and poor interobserver agreement on phenotypic designation. Radiographic features of RAS led to the highest interobserver agreement and were strongly associated with death or re-transplant [33]. This study highlights the limitations of CLAD diagnosis, particularly in single-lung transplant recipients, and acknowledges that CLAD remains a clinical diagnosis reliant on the diagnostician’s acumen.

### 3.2. CLAD Management

There is a growing body of published literature showing effective treatment strategies for CLAD. Unfortunately, response to therapy is variable, with many patients continuing to progress despite intervention. Many of these interventions focus on modifying or augmenting immunosuppression, immunomodulation, and mitigating risk factors for progressive lung injury. We will review some of the most frequently studied interventions employed in the management and prevention of CLAD.

#### 3.2.1. Calcineurin Inhibitors (CNIs)

As treatment options and efficacy are limited for CLAD, prevention of allograft dysfunction is of utmost importance. CNIs are a mainstay of lung transplant maintenance immunosuppression. There have been multiple randomized controlled trials (RCTs) demonstrating tacrolimus to be the superior CNI when compared to cyclosporine in preventing the development of CLAD. Treede et al. showed in a multi-center, prospective, randomized controlled trial that in 249 patients, the cumulative incidence of CLAD at three years was 11.6% for tacrolimus compared to 21.3% with cyclosporine (*p* = 0.037, hazard ratio [HR] 1.97 [95% CI 1.04 to 3.77], *p* = 0.039) [34]. Dellgren et al. conducted an open-label, multi-center, randomized control trial with 249 patients, which showed that once-daily tacrolimus compared to twice-daily cyclosporine significantly reduced the incidence of CLAD at three years (13% vs. 39% (hazard ratio [HR] 0.28 [95% CI 0.15–0.52], log-rank *p* < 0.0001) [35]. A systematic review published in 2024 with four RTCs, which included the two above studies and included an additional two studies with 164 additional patients, again showed decreased risk of CLAD development with tacrolimus use instead of cyclosporine [36]. These outlined trials represent some of the few multi-center RTCs performed to study management and prevention of CLAD.

#### 3.2.2. Cell-Cycle Inhibitors

Mycophenolate (MMF) and azathioprine (AZA) are antimetabolites that are used in maintenance immunosuppression in lung transplant recipients alongside CNIs and corticosteroids. In a small, single-arm study of 13 patients with CLAD, changing to MMF from AZA resulted in PFT stabilization in a majority of patients with CLAD [37]. Speich et al. prospectively observed 108 patients at a single center treated with MMF and 48 with AZA, and while there was no change in incidence of BOS, there was a significant decrease in incidence of graft loss (*p* = 0.049) and a trend towards improved survival (*p* = 0.062) [38]. Notably, 42% of the patients initially treated with AZA were changed to MMF after a median of 1169 days because of recurrent acute rejection or BOS [38]. McNeil et al. conducted an open-label, randomized trial of 156 lung transplant recipients for three years comparing MMF and AZA and found no difference in the incidence, time to onset, or severity of CLAD [39]. Similarly, Palmer and colleagues prospectively randomized 81 patients from two centers to receive AZA (n = 38) vs. MMF (n = 43), and there was no difference in acute rejection or survival rates at 6 months [40]; the incidence of CLAD was not obtained, and follow-up was only for the initial six-month post-transplant period. There is variable data on mycophenolate use compared to azathioprine, but with an overall trend towards favoring MMF over AZA for prevention of ACR [41], CLAD, and a small amount of data favoring improved lung function.

#### 3.2.3. Surgical Management of Gastroesophageal Reflux

Prevention of CLAD is an important goal, as treatment of CLAD is difficult and inconsistent. GERD is prevalent in lung transplant recipients, worsens after transplant [42,43], worsens lung function, and is a risk factor for CLAD [11,44,45,46]. Medical management alone for GERD does not address non-acid reflux and is of limited utility in preventing CLAD [47,48,49]. Hartwig and colleagues in a single-center trial prospectively found that of 157 of 222 (53%) patients with reflux who had a fundoplication within the first year of transplant, predicated peak and one-year FEV1 were significantly better in those who had anti-reflux surgery [49]. Davis et al. reported on 26 patients who initially met CLAD criteria prior to fundoplication for GERD, and after fundoplication, 16 patients had improvements, with 13 no longer meeting CLAD criteria [50]. Similar observational studies also found a role for fundoplication in improved FEV1 and prevention of CLAD [10,51,52], and as such, some centers elect to perform pre-emptive anti-reflux surgeries in selected transplant recipients to mitigate risk.

#### 3.2.4. Azithromycin

Azithromycin, a macrolide antibiotic, has been utilized in a variety of clinical contexts to take advantage of immunomodulating effects in lung tissue, including COPD and graft-versus-host disease following bone marrow transplant. It comes as no surprise that it has also been explored in the management of CLAD. Vos and colleagues randomized 83 patients post-transplant to receive either azithromycin or a placebo and followed them prospectively for two years to determine CLAD occurrence. The incidence of CLAD was 12.5% versus 44.2% (*p* = 0.0017), and CLAD-free survival was better with azithromycin (HR 0.27, [95% CI 0.092–0.816]; *p* = 0.020) [53]. They also prescribed open-label azithromycin for those who developed CLAD, and FEV1 improved in 52% of patients. Corris et al. conducted a randomized placebo-controlled trial of 48 patients with CLAD who were given azithromycin 250 mg on alternating days vs. placebo for 12 weeks and compared FEV1 measurements at 12 weeks. They found an estimated mean difference of 0.278 L in FEV1 in patients with azithromycin ([95% CI 0.170–0.386 L], *p* =< 0.001) [54]. Smaller studies have found similar improvement in FEV1 with initiation of azithromycin for CLAD [55]. Kingah et al. performed a meta-analysis of 10 studies looking at azithromycin’s effects on FEV1 change in patients with CLAD. One hundred and forty patients were treated for an average of seven months, and the mean percentage increase in FEV1 was 8.8% (CI 5.1–12.47, *p* < 0.001), with a hazard ratio of 0.25 (CI 0.06–0.56, *p* = 0.041) [56]. Patients with pulmonary dysbiosis and high microbial burden may have a more favorable response to azithromycin therapy, including improved survival [57]. Related to this, airway neutrophilia may predict response to azithromycin in CLAD [58]. There is considerable practice variability on when to introduce azithromycin, with almost a third of centers initiating azithromycin prophylactically during transplant hospitalization, and 14% initiating at the time of CLAD diagnosis. Many centers initiated azithromycin following recognition of a CLAD event, and optimal timing of initiation remains unclear [59].

#### 3.2.5. Montelukast

Montelukast is a leukotriene inhibitor potentially useful in CLAD due to decreasing eosinophil-driven inflammation [18]. Ruttens et al. conducted a prospective, randomized, double-blind, placebo-controlled trial in 30 CLAD patients. Patients with a rapid decline in FEV1 (>150 mL/month) or those within two years of transplant were excluded. There was no difference in graft loss or lung function, but a post-hoc analysis found slowing of the rate of FEV1 decline in CLAD stage 1 patients given montelukast compared to placebo, though there were only 11 and eight patients, respectively, in each arm [60]. Vos and colleagues retrospectively evaluated 152 patients from a single center with progressive CLAD despite three months of azithromycin, 115 (75%) with BOS, and 38 (25%) with RAS, who were given montelukast for at least three months. Montelukast was associated with an attenuated rate of FEV1 decline at three and six months (both *p* < 0.0001), independent of CLAD stage [61]. Those with lung function who stabilized or improved also had a significantly better progression-free (*p* < 0.0001) and overall survival (*p* = 0.0002) compared to those who had further FEV1 decline [61].

#### 3.2.6. mTOR Inhibitors

Everolimus and Sirolimus are inhibitors of mammalian target of rapamycin (mTORi) and have been used in conjunction with maintenance immunosuppression to minimize CNI nephrotoxicity [62]. A retrospective review of 57 patients with CLAD who were started on everolimus with a 50% reduction in tacrolimus troughs [63] was performed; 56.2% had BOS 0-p or stage 1, while 33.2% had BOS stage 2 or 3, and, notably, only three (5%) patients had RAS, limiting analysis of this subgroup. BOS patients demonstrated improvements in the rate of decline in FEV1, from −102.7 mL/month in the six months prior to everolimus initiation to −7.4 mL/month 12 months post-initiation (*p* < 0.05). The FEV1 improvement was seen in both early- and later-stage BOS patients, but a benefit was most pronounced in individuals with BOS stage 0 or stage 1 [63]. Notably, everolimus was withdrawn in 22 patients (38.6%) due to edema, leukopenia, or progression of CLAD. Another retrospective study analyzed absolute percent-predicted FEV1 change in 17 patients with CLAD (seven patients with RAS and ten with BOS) at 12 months post everolimus introduction [64]. They found stabilization of percent-predicted FEV1 (46.7% to 51.0%) in BOS patients, with a decline in the percent-predicted FEV1 (59.2% to 44.2%) in patients with RAS [64]. Despite the small sample size, this study highlights the nuances of managing CLAD and differential response to therapy based on phenotype. Sacher and colleagues performed a retrospective review and found 19 of 24 patients tolerated long-term mTORi for maintenance immunosuppression, and when compared to 22 non-mTORi-treated comparators, the mTORi group had a significantly lower incidence of CLAD (*p* = 0.05), and higher survival rates (*p* = 0.004) followed for a median of 6.9 years [65]. Other studies have been less conclusive, with Glanville and colleagues observing no difference in the development of CLAD at three years between everolimus and mycophenolate [66]. Transplant programs vary widely in how and why they initiate mTOR inhibitors. Typical indications include reducing CNIs to lessen nephrotoxicity and neurotoxicity, along with potential antineoplastic and anti-cytomegalovirus effects. Additionally, there is a significant discontinuation rate, which can impact the reliability of studies, as many are analyzed as intention-to-treat. Tolerance may be limited due to adverse effects, including headaches, edema, poor wound healing, and endocrinopathies. Additionally, mTOR inhibitor initiation must be delayed following transplant to permit healing of the anastomoses.

#### 3.2.7. HMG-CoA Reductase Inhibitors (Statins)

Consideration has been given to the use of HMG-CoA reductase inhibitors (statins) as a preventative strategy for CLAD due to anti-inflammatory and immunomodulatory effects, including major histocompatibility class II inhibition [67]. Data on statin use have been conflicting, with some studies suggesting a statistically significant reduction in the rate of CLAD [68,69,70], while others show no difference [71]. However, in a single-center retrospective study that did not show a difference in CLAD development with statin use, Szczpanik and colleagues demonstrate a reduction in the incidence of CLAD-related death and a survival benefit [71]. Variability in the timing of drug initiation and evolving CLAD classifications may be confounding study results. Dyslipidemia and cardiovascular disease are frequently observed comorbidities in lung transplant recipients, and, as such, many patients already have an indication to initiate statin therapy.

#### 3.2.8. Anti-Thymocyte Globulin

Anti-thymocyte globulin (ATG) is a polyclonal antibody derived from horses or rabbits immunized to human T-cells and induces weeks- to months-long depletion of T-cells, B-cell apoptosis, interference with dendritic cell function, and induction of regulatory T-cells and natural killer T-cells [72]. Multiple retrospective, single-center studies have evaluated the ATG in patients with CLAD (Table 1). Izhakian et al. studied 25 patients with CLAD who received ATG. Eight (32%) patients had stabilization of their FEV1 and lived longer than the 68% of patients who did not stabilize after ATG administration [73]; notably, those who responded had a slower pre-treatment decline in FEV1. Dunn and colleagues retrospectively reviewed 63 patients given ATG. Of the cohort, 82.5% had a BOS phenotype, 68.3% had stage 1 or 2 CLAD, and 69.8% qualified as “rapid decliners”, with a FEV1 decline of greater than 100 mL/month; the average rate of decline prior to ATG administration was 5.0 mL/day. They found 49 patients (77.8%) had at least a 20% attenuation of the rate of FEV1 decline; however, a minimal number of patients had increased FEV1 (12.7%) [74]. Kotecha et al. also found a similar proportion of “responders” with ATG and CLAD [75]. Forty-five of 71 (63%) patients, 87% with a BOS phenotype, had an improvement in mean FEV1 rate of decline from 6.1 to 1.6 mL/day [75]. January et al. included 108 patients with CLAD who received ATG; again, all but seven (6.5%) patients had a BOS phenotype. After treatment, 43 patients (40%) had an increase in FEV1, while 47 (43.5%) had slowed the FEV1 rate of decline [76]. Padhye et al. evaluated 124 patients with CLAD, 55 (44%) who received ATG and 69 (56%) who did not; 67.7% of the patients had a BOS phenotype. ATG therapy was not associated with a significant difference in the rate of FEV1 decline between the two groups, but the one-year mortality post-CLAD diagnosis was 50% for people who did not receive ATG and 35.5% for those who did [77]. ATG may stabilize graft function and provide a window of opportunity for other interventions, such as fundoplication, optimization of maintenance immunosuppression, or evaluation for re-transplantation.

#### 3.2.9. Alemtuzumab

Alemtuzumab is a monoclonal antibody against CD52, which is expressed mainly on B and T lymphocytes, and induces a profound and prompt lymphocyte depletion that can last for many months. Reams et al. performed a single-center observational cohort study of 10 patients with CLAD alemtuzumab after progression with ATG and prednisone. They observed improvement or stabilization of BOS grade in 70% of patients [78]. Ensor and collaborators found freedom from BOS progression in 53% of the 17 patients given alemtuzumab. There was no FEV1 decline in 70% of early-stage CLAD patients, though this benefit was only observed 14% in advanced CLAD patients [79]. Due to extensive and prolonged lymphopenia and depressed CD4 counts, which must be monitored closely following administration, broad infection prophylaxis is usually employed in patients who have received alemtuzumab until count recovery. Despite this, there were significant infection rates in these studies (73% and 73.7%) [78,79]. Alemtuzumab appears to have some benefit in slowing down CLAD progression, but this should be balanced with risks, including infection, malignancy, and bone marrow suppression.

One potential consideration is the use of alemtuzumab as induction therapy and its subsequent role in CLAD-free survival. Peri-operatively, patients receive intensive immunosuppression, with most centers using interleukin-2 receptor antagonists such as basiliximab or daclizumab (prior to discontinuation), or alternatively, ATG [80]. Some centers have pivoted to utilizing alemtuzumab as their induction therapy, rapidly and durably depleting lymphocytes. Benazzo and colleagues demonstrated longer freedom from CLAD in their single-center retrospective cohort of 721 patients, with five-year CLAD-free survival at 72% [81] compared to the international standard of approximately 50% [2]. They saw low rates of both infection and malignancy in their cohort; these are frequently cited as concerns regarding alemtuzumab administration. An earlier retrospective single-center cohort of 336 patients, 127 of whom underwent induction therapy with alemtuzumab, demonstrated greater five-year CLAD-free survival when compared to ATG, daclizumab, or no induction therapy [82].

#### 3.2.10. Extracorporeal Photopheresis (ECP)

Extracorporeal photopheresis (ECP) is a technique that combines leukapheresis with photoactivation. It tags mononuclear cells with 8-methoxypsoralen and exposes the cells to ultraviolet radiation, which induces apoptosis of T-cells, potentially stabilizing graft function. Most studies are limited to small, single-center cohorts. Jaksch and colleagues performed a single-center prospective study, which showed that 31 of 51 (61%) patients with CLAD treated with ECP showed six-month stabilization of lung function; they also compared CLAD patients who received ECP treatment with those who did not and saw an improved graft survival [83]. Similarly, other studies showed that a majority of patients stabilized or improved lung function with ECP initiation for CLAD [84,85,86]. Recently, Benazzo and colleagues performed a retrospective analysis of a large multi-center cohort of 613 European lung transplant recipients. Of these patients, a majority (90%) had received azithromycin therapy, and a third had also trialed montelukast; despite this, their disease continued to progress. Long-term stabilization of graft function was achieved in 53% of the cohort, 10% showed an improvement, while the remaining 37% fail to respond to ECP; stable patients and responders were predominantly BOS patients, while non-responders were mostly RAS (*p* = 0.005) [87]. As seen in prior studies outlined above, this highlights the differential response to therapy amongst CLAD phenotypes, which poses a challenge to management.

Moniodis et al. retrospectively compared the efficacy of ECP (n = 17) to alemtuzumab (n = 13) for treatment of progressive CLAD at a single center. Both groups had a significant improvement in FEV1 rate of decline at three and six years, and similar rates of infectious complications and survival at one year [88]. Of interest, a third cohort of slowly progressing CLAD patients who received no immunomodulatory treatment was studied; these patients had a more gradual rate of FEV1 decline when compared to patients who went on to receive alemtuzumab or ECP. The post-treatment FEV1 decline trends in both ECP and alemtuzumab-treated arms closely approximated the no-treatment, slow-progression arm, suggesting that these therapies may shift phenotypes from rapid to gradual progression of CLAD. This represents one of the rare studies that sought to compare two immunomodulatory therapies for chronic lung allograft dysfunction.

While ECP does have evidence demonstrating graft stabilization in some patients, the practicality of this intervention limits its implementation, due to access to apheresis centers, cost, and time commitment on behalf of the patient.

#### 3.2.11. Total Lymphoid Irradiation

Total lymphoid radiation (TLI) is radiation that targets certain radiosensitive B-cell and T-cell populations, allowing a shift towards a high regulatory T-cell endotype [89]. Studies overall show a slowing in FEV1 decline in patients with CLAD, regardless of phenotype or rapid FEV1 decline [90,91,92]. Fisher et al. studied 37 lung transplant recipients retrospectively from a single center, the majority of whom had BOS stage 2 or 3 (81%). Twenty-seven people (73%) completed at least eight of 10 fractions of radiation. Of the 27 recipients who completed treatment, FEV1 decline slowed from 122.7 mL/month to 25.1 mL/month (*p* = 0.0004) [90]. Ten people (27%) did not complete treatment, two because of death from progressive CLAD and eight from TLI complications, including infection (two people) and bone marrow suppression (six people). Lebeer and colleagues retrospectively reviewed 20 patients, 19 of whom had BOS stage 2 or 3, who received TLI. The FEV1 decline decreased from 196 mL/month to 59 mL/month six months pre- to six months post-treatment (*p* = 0.0002) [92]. Temporary discontinuation of TLI occurred in 5/20 (25%) of patients due to leukopenia, and two patients (10%) died during TLI treatment due to progressive CLAD. Geng-Cahuayme et al. retrospectively evaluated their data on 40 patients with CLAD (29 with BOS, nine with RAS, two with mixed phenotype) and saw a decrease in the slope of FEV1 decline from −78 mL/month to +2 mL/month. There was no difference in improvement between the BOS and RAS groups, and TLI was not discontinued in any patients due to toxicity [91]. One study suggested that the attenuation in graft loss may be more profound in individuals who experience a rapid decline in lung function [93]. Overall, compared to other therapies, TLI is less frequently employed when compared to other advanced therapies, and center experience is widely variable.

#### 3.2.12. Antifibrotic Therapy

Antifibrotic therapies such as pirfenidone and nintedanib have been used in idiopathic pulmonary fibrosis (IPF) to slow the loss of FVC over time in the INPULSIS and ASCEND trials [94,95]. Postulating a similar benefit to the scarring in CLAD, and especially RAS, a case series and some case reports have looked at the antifibrotics in CLAD. A small retrospective analysis of 11 patients with CLAD, RAS phenotype, who took pirfenidone for at least three months, demonstrated some promise. Three of the eleven patients demonstrated graft stability, three were bridged to re-transplant, and five died. There was a significant need for dose de-escalation (55%) due to poor tolerance. The authors concluded that pirfenidone may attenuate the rate of decline of lung function in patients living with RAS [96]. Other case series conflict on the efficacy of RAS improvement with nintedanib and pirfenidone [97,98]. Unfortunately, one of the more promising studies on this front, STOP-CLAD, did not meet enrollment goals due to the SARS-CoV-2 pandemic; 23 of a planned 60 patients in this randomized, single-center study were included in the analysis [99]. There was no significant difference with regard to progression of radiographic allograft dysfunction or lung function decline among the studied participants, and, at the time, represented the largest cohort of patients. The authors did acknowledge that a more targeted study investigating the role of the RAS phenotype may be warranted, given the overlapping features with pulmonary fibrosis [99]. Subsequent to this, Perch and colleagues published findings of an international, randomized, double-blinded, multi-center controlled phase II trial evaluating bilateral lung transplant recipients with progressive CLAD randomized to pirfenidone or placebo [100]. This included 90 patients, with 48 randomized to pirfenidone and 42 to placebo, for 26 weeks. There was considerable premature discontinuation, with 59 of the 90 patients completing the six-month follow-up. There was no statistically significant difference in the rate of decline in lung function, graft loss, death, or re-transplantation among study groups [100].

#### 3.2.13. Re-Transplantation

While re-transplantation can be a consideration for patients with CLAD unresponsive to other therapies, patient selection must be carefully considered. Harhay et al. conducted a retrospective cohort study of first-time retransplanted lung recipients from the ISHLT Registry data from May 2005 through June 2017 [101]. The cohort included 1597 patients, with 43% being retransplanted for CLAD. One-year survival was 76% and 69% for double and single-lung recipients, respectively, much lower than the 88.4% combined first-time double- and single-lung transplant one-year mortality [102]; notably, re-transplantation for CLAD vs. non-CLAD indications was a positive prognostic factor (*p* < 0.0001) [101]. Two other studies found retransplant to be associated with lower peak lung function and a higher post-operative mortality, particularly in patients with RAS and less than two years from primary transplant [103,104]. Unfortunately, in many studies, treatment response in the RAS phenotype of CLAD is worse than that of patients with BOS [24,105].

## 4. Discussion and Future Directions

Recent consensus guidelines from the ISHLT recommend that future studies investigating CLAD with regard to the most relevant endpoints for trials [105]. For CLAD prevention trials, they recommend using a primary endpoint of time from initiation of study intervention to timing of CLAD onset, while considering death and re-transplant. CLAD treatment trials should evaluate time from treatment assignment to time of CLAD progression (preferentially utilizing decline in FEV1), with death and re-transplant included as composite endpoints. CLAD has been associated with reduced quality of life and performance status, so therapies that delay or slow down the progression of CLAD are likely to delay decrements in quality of life and performance status.

Future areas of interest include identification and performance characteristics of biomarkers as a non-invasive method to detect CLAD or identify individuals at risk for CLAD who may be eligible for early interventions. Over the past decade, there has been increasing traction in how to use donor-derived cell-free DNA (dd-cfDNA) in identifying graft dysfunction (AMR, ACR, CLAD). When donor allograft cells are damaged, they release DNA into the circulation with a unique footprint distinct from recipient DNA. Several studies have evaluated the use of dd-cfDNA in identifying patients at risk for CLAD with some promising results [106,107], although, at present, there are no clear guidelines or widely adopted methods on how to utilize this data. Other circulating biomarkers of interest include identification of auto-antibodies, which are not yet commercially available, assessment of telomere length, and glycoproteins expressed by pulmonary epithelial cells [108,109]. Radiologic assessment of lung volumes, density, and assessment of pulmonary vasculature may enhance current radiographic tools used to diagnose CLAD [110,111,112,113]. Augmented intelligence may provide another unique opportunity in the assessment and characterization of CLAD, both in image assessment and in the interpretation of samples, such as bronchoalveolar lavage fluid and eNose [114,115]. Further insight into the lung microbiome may provide a more nuanced assessment of CLAD diagnosis, prognosis, and treatment [57]. The introduction of such tools provides unique opportunities for translational studies to advance diagnosis (and, therefore, treatment) of CLAD, including in patients that have been historically challenging to diagnose, such as single-lung transplant recipients.

Even though there are a few high-quality, prospective, placebo-controlled studies showing effective treatment of CLAD, many of the above medications and therapies are commonly employed. There are some less-established and emerging therapeutics as well. Inhaled cyclosporine has mixed results on improvement in CLAD-free survival [116,117]. Methotrexate has been shown in small studies to slow the rate of FEV1 decline in patients with CLAD [18]. While the de novo introduction of belatacept had increased mortality in a pilot study, outcome data regarding the use of belatacept as a CNI-sparing regimen pertaining to CLAD have yet to be reported. Further complicating research in CLAD therapeutics are the various phenotypes of CLAD, with RAS-type displaying a shorter transplant-free survival and less consistent response to therapy, making it particularly challenging to address; given the relatively low incidence of RAS, it also makes large, well-designed trials focusing on this phenotype more difficult to perform.

In addition to improved quality of data for existing CLAD therapeutics, there are many additional treatment options to explore. There are similarities between CLAD and pulmonary graft vs. host disease (GvHD), and pulmonary GvHD therapies such as tyrosine kinase inhibitors, janus kinase inhibitors, and rho kinase inhibitors could be evaluated in CLAD [118,119]. Lung bioengineering and gene therapy utilizing ex vivo lung perfusion, prior to implantation, may also provide an opportunity to intervene on CLAD before the transplant has occurred [120]. Further understanding of the genetic and immunologic profiles among different patients with CLAD could also elucidate novel biological targets for treatment.

CLAD remains a formidable challenge in the world of lung transplantation and a major threat to a patient’s survival and quality of life, and is multifaceted in its diagnosis and management. The current diagnostic guidelines [23] have provided a framework for diagnosing, phenotyping, and staging CLAD that was lacking. Clinical trials evaluating the safety and efficacy of CLAD-directed therapies are limited by small sample sizes, single-center studies, or retrospective reviews in many cases, and variability in practice patterns between transplant centers leads to significant heterogeneity with regard to the management of CLAD. There is a paucity of large, randomized trials evaluating management of CLAD, due in part to heterogeneity in practice patterns amongst transplant centers, as well as the overall low incidence of lung transplant, and therefore CLAD, within the population. There remains an emphasis on early management of risk factors, including infection and gastroesophageal reflux, timely detection and characterization, and thoughtful application of current therapies in an effort to stabilize graft function.

Reversal of graft loss is rare with treatment, and most therapies aim at either stabilizing graft function or slowing down the rate of decline in CLAD. Despite the above articulated therapies, many times treatment fails. Transplant providers have to be conscientious of this and have thoughtful conversations with patients regarding anticipated outcomes and prognosis.

## Figures and Tables

**Table 1:** Summary of selected trials evaluating ATG in the treatment of CLAD.

Authors	Population	Outcome
Izhakian et al. (2016) [73]	Twenty-five LTx recipients with CLAD who received RATG therapy between May 2005–February 2016.	Response divided into stabilization of lung function (8/25, 38%) and ongoing decline (17/25, 68%). Stabilization group demonstrated longer survival after RATG (930 ± 385 days compared to 414 ± 277 days).
Dunn et al. (2024) [74]	One-hundred-and-thirty-six LTx recipients between 2010–2022, 72 of whom received ATG for CLAD (63 in final analysis) and 64 of whom did not.	Of 63 RATG recipients, 77.8% had at least a partial response to ATG (>20% attenuation in rate of FEV1 decline), 12.7% had complete response (stabilization or improvement in FEV1 after therapy), and 22.2% did not respond to RATG. Infection and malignancy were frequent complications following RATG administration.
Kotecha et al. (2021) [75]	Seventy-six LTx patients (January 2006–December 2017) including bilateral, single, and re-transplant recipients who underwent first-line CLAD therapy with methylprednisolone. Excluded five patients who had a clinical diagnosis of antibody-mediated rejection.	Sixty-three percent of patients included were clinical responders, including 23% complete responders (absolute improvement or stability in FEV1) and 40% partial responders (rate of FEV1 decline improving by 20% or more). Responders had improved retransplant-free survival.
January et al. (2019) [76]	One-hundred-and-eight LTx recipients included in final analysis, diagnosed with CLAD > 6 mo out from transplantation, who received RATG as first-line therapy between January 2009–June 2016. Excluded patients with ACR, positive cultures for pathogenic organisms, prior ATG therapy, or previously received photopheresis or antibody desensitization therapy.	Forty-three patients (40%) had an increase in FEV1 compared to pretreatment measures, classified as responders. 65 (60%) of patients had a further decrease in FEV1. Of non-responders, 47 (43.5%) had a less negative slope in FEV1 in the six months after treatment, indicating slower decline in lung function. Serum sickness (24 patients, 22.2%) and infectious complications (20 patients, 18.5%) were the most-reported adverse reactions.
Padhye et al. (2025) [77]	One-hundred-and-twenty-four LTx recipients with progression of CLAD despite initial treatment, including single, double, and re-transplant recipients. Of this cohort, 55 were treated with RATG.	No significant difference in the rate of FEV1 decline amongst groups, but one-year cumulative mortality trended lower in the ATG group (35.5%) compared to the no-ATG group (50%) (aHR 0.66 [0.39–1.14], *p* = 0.134). Authors suspect it may be related to bias not accounted for in study design, or protective effects of ATG against other sequelae of CLAD.

Note that all studies included are single-center, retrospective analyses.

## Data Availability

No new data were created or analyzed in this study.

## Abbreviations

ACR	acute cellular rejection
AMR	antibody mediated rejection
ATG	anti-thymocyte globulin
AZA	azathioprine
BOS	bronchiolitis obliterans syndrome
CNI	calcineurin inhibitor
CLAD	chronic lung allograft dysfunction
ddCF-DNA	donor derived cell-free DNA
DSA	donor specific antibody
EVLP	ex vivo lung perfusion
FEV1	forced expiratory volume at 1 s
FVC	forced vital capacity
ISHLT	International Society for Heart and Lung Transplantation
IPF	idiopathic pulmonary fibrosis
LTx	lung transplant
mTOR	mammalian target of rapamycin
PFT	pulmonary function testing
PPF	progressive pulmonary fibrosis
RAS	restrictive allograft syndrome
TLC	total lung capacity
TLI	total lymphoid irradiation
